# A Study of Applying Pulsed Remote Field Eddy Current in Ferromagnetic Pipes Testing

**DOI:** 10.3390/s17051038

**Published:** 2017-05-05

**Authors:** Qingwang Luo, Yibing Shi, Zhigang Wang, Wei Zhang, Yanjun Li

**Affiliations:** 1 Center for Information Geoscience, University of Electronic Science and Technology of China, Chengdu 611731, China; qwluo@std.uestc.edu.cn (Q.L.); weizhang@uestc.edu.cn (W.Z.); yjli@uestc.edu.cn (Y.L.); 2 School of Automation Engineering, University of Electronic Science and Technology of China, Chengdu 611731, China; wangzhigang@uestc.edu.cn

**Keywords:** PRFECT, ANSYS software, LSSVR, zero-crossing time, double sensing coils, Wiener deconvolution filter, zero-mean normalization

## Abstract

Pulsed Remote Field Eddy Current Testing (PRFECT) attracts the attention in the testing of ferromagnetic pipes because of its continuous spectrum. This paper simulated the practical PRFECT of pipes by using ANSYS software and employed Least Squares Support Vector Regression (LSSVR) to extract the zero-crossing time to analyze the pipe thickness. As a result, a secondary peak is found in zero-crossing time when transmitter passed by a defect. The secondary peak will lead to wrong quantification and the localization of defects, especially when defects are found only at the transmitter location. Aiming to eliminate the secondary peaks, double sensing coils are set in the transition zone and Wiener deconvolution filter is applied. In the proposed method, position dependent response of the differential signals from the double sensing coils is calibrated by employing zero-mean normalization. The methods proposed in this paper are validated by analyzing the simulation signals and can improve the practicality of PRFECT of ferromagnetic pipes.

## 1. Introduction

Ferromagnetic pipes are largely used in the transportation and exploration of oil and gas, and their monitoring and prevention of corrosion is highly important [1,2,3]. Remote Field Eddy Current Testing (RFECT) is primarily interesting because of its advantages, such as equal sensitivity to inner and outer defects, insensitivity to lift-off effects and contactless testing [4,5]. In order to overcome some inherent drawbacks of RFECT [4,5], such as the single spectrum, long probes, weak testing signal, and so on, studies are further focused on applying Pulsed Remote Field Eddy Current Testing (PRFECT) in ferromagnetic pipes [6,7,8,9,10]. A majority of these studies are performed in time domain [6,7,8,9,10], and the voltage magnitude and the zero-crossing time are extracted to analyze the effects of pipe inner diameter and pipe thickness, respectively. 

In order to study the features of applying PRFECT in ferromagnetic pipes, the authors simulated the practical PRFECT of pipes by using ANSYS software. In the simulation, the transmitter and sensing coil moved simultaneously with the step of 10 mm, only one defect was built, and the simulation was performed 100 times. Because the pipe thickness affects the value of the zero-crossing time [6,7,8,9,10], in the signal processing, the zero-crossing time of every testing signal was extracted by employing Least Squares Support Vector Regression (LSSVR) [11,12] to analyze the pipe thickness, and came to the conclusion that, in the zero-crossing time, a secondary peak appeared when the transmitter passed by the defect. The secondary peak appeared in practical PRFECT of pipes is nearly not studied all over the world, even though the secondary peak leads to a wrong assessment of the defect, including the quantification and the localization. This paper studies the secondary peaks in PRFECT of ferromagnetic pipes, and provides methods to eliminate the secondary peaks. According to the authors’ studies on RFECT in ferromagnetic pipes [13,14,15], double sensing coils are set in the transition zone and the Wiener deconvolution filter is used to remove the secondary peak. In the paper, the errors caused by the double sensing coils are also discussed and calibrated. This paper provides methods to process practical problems of PRFECT of ferromagnetic pipes and they are verified by analyzing the simulation signals.

## 2. Methods and Models 

### 2.1. LSSVR

**Figure 1:**
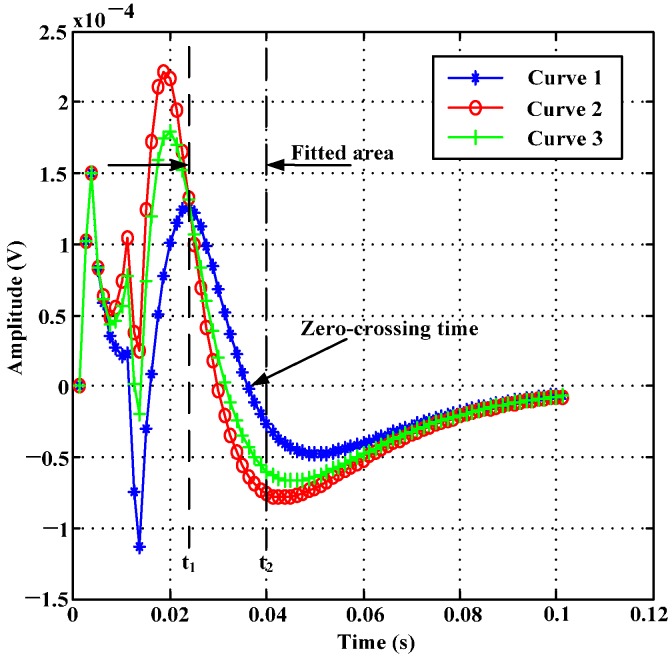
Schematic layout of the fitted area nearby zero-crossing time: Curve 1 is the induced signal when both sensing coil and transmitter are placed non-defect area; Curve 2 is the induced signal when only sensing coil is placed defect area; and Curve 3 is the induced signal when only the transmitter is placed defect area.

Because of the good approximation accuracy and generalization ability of Least Squares Support Vector Regression (LSSVR) [11,12], this paper used LSSVR to extract the zero-crossing time from simulation signals to analyze the wall thickness feature of practical PRFECT in ferromagnetic pipes. In order to ensure the uniqueness of the zero-crossing time extracted from a simulation signal and to simplify the inversion model, only parts of a simulation signal are used to fit the inverse function, as shown in Figure 1.

As shown in Figure 1, the zero-crossing time is between t_1_ and t_2_. In order to obtain the zero-crossing time of every simulation signal, the inversion model built with LSSVR [11,12] can be described as follows,

(1) $$f(x)=\sum_{i=1}^{m}\alpha_{i}\kappa (x,x_{i})+b$$

where f(x) is the inversion time, $\kappa (x,x_{i})$ is the Radial Basis Function (RBF), $x_{i}$ is the amplitude between t_1_ and t_2_, m is the number of amplitudes, and $\alpha_{i}$ and b are the coefficients. 

The procedures of obtaining f(x) are realized in MATLAB with the LSSVR toolkit, and f(0) is used to inverse the zero-crossing time of every simulation signal. The inversion procedures are operated 200 times, and it takes 40 s in total.

### 2.2. Testing Model

**Figure 2:**
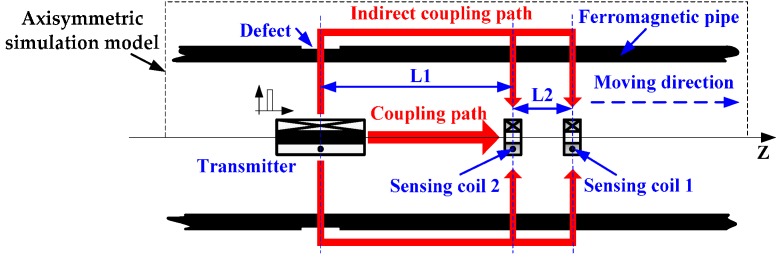
The model used for removing secondary peaks in Pulsed Remote Field Eddy Current Testing (PRFECT) of ferromagnetic pipes.

The zero-crossing time is extracted to analyze the features of pipe thickness in PRFECT of pipes by employing LSSVR, and a secondary peak is found when the transmitter passed by defects. To remove the secondary peak, double sensing coils are set in transition zone, as shown in Figure 2.

In Figure 2, sensing coil 1 and sensing coil 2 are both set in the transition zone, and they are made of the same material and are the same sizes. The transmitter is employed to excite a pulsed signal at a low frequency (0–100 Hz). Pulsed signal is regarded as a superposition of sinusoidal signals with different frequencies. Wall thickness affects these sinusoidal signals following the well-known “skin-effect” Equation [6],

(2) $$A=A_{0}e^{-d\sqrt{\pi f\mu \sigma }}\sin(2\pi ft-d\sqrt{\pi f\mu \sigma })$$

where A is the induced signal at any depth of the sample, $A_{0}$ is the amplitude of excited signal, f is the frequency of excited signal, d is the distance of the signal penetrated, t is signal time, and μ and σ are the permeability and conductivity, respectively.

According to Equation (2), at any time ( t ), the influence of a defect on the amplitude of a sample can be given by,

(3) $$A_{c}=A_{0}e^{-(d-\Delta d)\sqrt{\pi f\mu \sigma }}$$

where Δd is the thickness of a defect.

Taking the logarithm of both sides of Equation (3), and transforming,

(4) $$\ln A_{c}=\ln A_{0}e^{-d\sqrt{\pi f\mu \sigma }}+\Delta d\sqrt{\pi f\mu \sigma }$$

According to Equation (4), the influence of a defect on the amplitude of a sample exhibits a superimposed effect. Based on Equation (4), the influence of defects nearby the transmitter on the testing signals from double sensing coils can be described as follows,

{(5a)A1(T)=A1′(T)+ΔA(T)(5b)A2(T)=A2′(T)+ΔA(T)

where $A_{1}(T)$ and $A_{2}(T)$ are the testing signal from sensing coil 1 and sensing coil 2, respectively, ΔA(T) is the influence of defects nearby the transmitter, and T indicates the testing time.

Because at the testing place T+ΔT , the sensing coil 2 has moved to where sensing coil 1 was ΔT ago, Equation (6) is obtained,

(6) $$A_{2}\prime (T+\Delta T)=A_{1}\prime (T)$$

where ΔT=L2/v , and v is the moving speed of the transmitter and sensing coils.

After substituting Equation (6) into the first subformula of Equation (5) and subtracting the first subformula from the second subformula of Equation (5), Equation (7) is obtained.

(7) $$A_{2}(T+\Delta T)-A_{1}(T)=\Delta A(T)*(h(T+\Delta T)-h(T))$$

where * is the convolution operation, and h(T) is the impulse function.

Based on Equation (7), Wiener deconvolution filter is employed to obtain ΔA(T) (details in [11,12,13]). After these processing, the influence of defects nearby the transmitter ( ΔA(T) ) can be removed from the testing signals.

### 2.3. Calibrations

Two calibrations should be made in Section 2.2. One calibration is made because the distance between the sensing coil 1 and transmitter is unequal to that between sensing coil 2 and transmitter. These unequal distances make Equation (6) invalid, and the zero-mean normalization method is used to eliminate this inequality, as shown in Equation (8).

(8) $$A_{i}\prime (T)=\left[A_{i}(T)-E(A_{i}(T))\right]/\sqrt{D(A_{i}(T))}$$

where $A_{i}(T)$ is the zero-crossing time extracted from the sensing coils, and i=1,2 indicate sensing coil 1 and sensing 2, respectively; $E(A_{i}(T))$ is the mean value of $A_{i}(T)$ ; and $D(A_{i}(T))$ is the variance of $A_{i}(T)$ .

Another calibration is made because of the factor h(T+ΔT)−h(T) in Equation (7). Aimnig to explain this, ΔA(T) is decomposed as in Equation (9).

(9) ΔA(T)=ΔA′(T)+N

where N can be any real constant and is irrelevant to testing time ( T ).

By substituting Equation (9) into Equation (7), Equation (10) is obtained as follows:

(10) $$\begin{array}{ll} A_{2}(T+\Delta T)-A_{1}(T) & =(\Delta A\prime (T)+N)*(h(T+\Delta T)-h(T))\\ & =\Delta A\prime (T)*(h(T+\Delta T)-h(T)). \end{array}$$

Equation (10) indicates that ΔA′(T) is the results obtained from Equation (7) and there exist a real constant ( N ) between ΔA′(T) and ΔA(T) . The real constant ( N ) can be compensated as follows:

1. By subtracting Equation (5b) from Equation (5a), Equation (11) is obtained as,
(11) $$A_{1}(T)-A_{2}(T)=A_{1}\prime (T)-A_{2}\prime (T)$$
2. By substituting Equation (6) into Equation (11), Equation (12) is obtained as,
(12) $$A_{1}(T)-A_{2}(T)=A_{1}\prime (T)*(h(T)-h(T-\Delta T))$$
$A_{1}\prime (T)$ can be used to indicate the defects nearby sensing coil 1.
3. Based on Equation (12), the Wiener deconvolution filter is applied to obtain $A_{1}\prime (T)$ .
4. Then N is computed by Equation (13),
(13) $$N=-\Delta A\prime (T_{j})$$
$T_{j}$ satisfies $A_{1}\prime (T_{j})\leq \left|\delta \right|$ , and δ is a constant approaches to zero.

The Steps 1 to 4 are realized in MATLAB, and several N are saved to obtain the mean value.

### 2.4. Simulation Sets

The model used to simulate practical PRFECT of ferromagnetic is built using ANSYS software. The simulation model is axisymmetric and 2D, as indicated in Figure 2 (the dotted portion). The amplitude of exciting pulse is 80 V, the repetition rate of excitation is 10 Hz, and the pulse duration is 10 ms. There is only one defect built on pipe wall, and its length and depth are 50 mm and 6 mm, respectively. The relative permeability of all coils is 1, and the relative permeability of pipe is 80. The other parameters of coils and ferromagnetic pipe set in ANSYS model are given in Table 1.

The range of the transition zone is usually within one times the inner diameter of testing pipe, and the distance between the two sensing coils should be less than one times the inner diameter when the length of every sensing coil is taken into consideration. The larger distance between the two sensing coils the better resolution for large area defects. However, large distance between the two sensing coils also increase the distance from the transmitter to the farther sensing coil, and this will enhance the power consumption.

The distance between middle of sensing coil 2 and middle of transmitter is 2.0 times the pipe inner diameter. Several distances between the middle of two sensing coils are simulated (0.2, 0.4 and 0.6 times the pipe inner diameter), and 0.4 times is chosen to present the theoretical validation of the proposed methods. However, in practical PRFECT of pipes, the distance between the middle of the two sensing coils should be chosen with the consideration of defect resolution, power consumption and pipe diameter range etc. The parameter values of the coils in this paper are obtained by optimizing the parameters of practical RFECT tool. In simulations, the transmitter and sensing coils moved simultaneously with the step of 10 mm. It takes nearly 25 min of each simulation on a computer with Intel(R) Core(TM) i5-4690 CPU@3.5GHz, 8.00 GB RAM, and simulations are operated 100 times.

## 3. Results and Discussion

**Figure 3:**
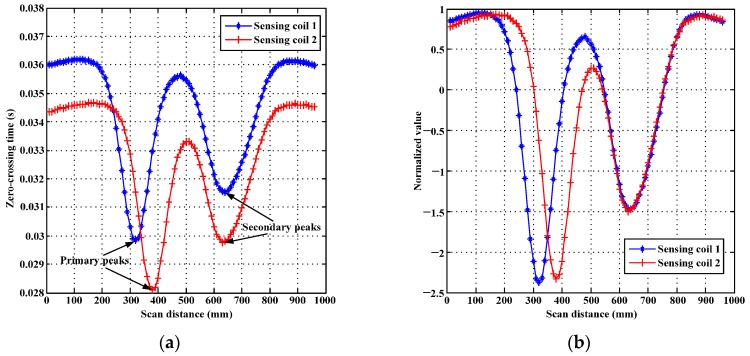
Demonstration of the extracted zero-crossing times: (**a**) the zero-crossing times directly extracted from the sensing coils by employing Least Squares Support Vector Regression (LSSVR); and (**b**) the calibrations made between zero-crossing times extracted from sensing coils 1 and sensing coils 2 using zero-mean normalization.

The results of using LSSVR to extract zero-crossing times from the induced signals on sensing coils are shown in Figure 3a. The calibrations of distance effect between the signals extracted from sensing coil 1 and sensing coil 2 by using zero-mean normalization are shown in Figure 3b.

As shown in Figure 3a, the zero-crossing time curve extracted from each sensing coil appears two peaks, even there is only one defect built in simulations. One peak appears when the sensing coil passed by the defect (primary peak), and the secondary peak arises when the transmitter passed by the defect. Figure 3a indicates that PRFECT has a same feature as the RFECT, and the feature reveals the secondary peak caused by the transmitter passing by defects. When the defects only occurred near the transmitter, wrong localization and quantification of defects will obtained because testing place (sensing coil place) has no defects. When the defects occurred at both locations of the transmitter and the sensing coils, the value of zero-crossing time (primary peaks) related to pipe thickness near the sensing coil is largely affected by the defects near the transmitter (which means the secondary peak are superimposed in the primary peak, please refer to [11,12,13] for more discussions about secondary peaks). The secondary peak that appears in Figure 3 will lead to a wrong localization and quantification of a defect, and its removal is necessary.

In order to remove the secondary peak from zero-crossing time, two sensing coils are used in the transition zone, and the calibration of the independent distances from sensing coils to transmitter is shown in Figure 3b. According to Figure 3b, the influence of the distance between the sensing coils and the transmitter is eliminated by applying zero-mean normalization method, and the scales of zero-crossing times extracted from the sensing coils are unified. 

**Figure 4:**
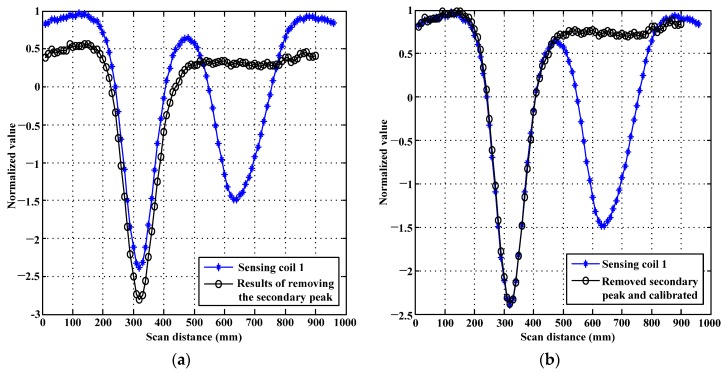
Schematic layout of the processing results by proposed method: (**a**) the comparison between the zero-crossing time and its removal of the secondary peak; and (**b**) the calibration of N .

This paper takes the signals from sensing coil 1 for instance. The results of removing the secondary peak are shown in Figure 4a, and the results of calibrating N are shown in Figure 4b. As shown in Figure 4a, the secondary peak is removed efficiently while the primary peak is maintained. Figure 4a validates the correctness of proposed method for removing the secondary peaks in PRFECT of pipes. It is apparent that there is a difference quantity between the two curves shown in Figure 4a. The difference quantity is the real constant ( N ) discussed in Section 2.3. By employing the method provided in Equations (11)–(13), the difference quantity ( N ) can be eliminated, as shown in Figure 4b.

## 4. Conclusions 

This paper investigates the features of applying Pulsed Remote Field Eddy Current Testing (PRFECT) in ferromagnetic pipes. In the investigation, the zero-crossing time is extracted using Least Squares Support Vector Regression (LSSVR) to analyze pipe thickness. As a result, the zero-crossing time exhibits the same feature as the signal in Remote Field Eddy Current Testing (RFECT) of pipes. The feature indicates that the secondary peaks are found when the transmitter passes by defects. The secondary peak appearing in the PRFECT of ferromagnetic pipes will lead to wrong localization and quantification of a defect, and its removal is essential. Further, double sensing coils are set in the transition zone to remove the secondary peaks, and the essential calibrations are also discussed and worked out. The methods proposed in this paper are validated by simulating in ANSYS software. Because the studies provided in this paper are an extension of authors’ research on RFECT of pipes, and the circuits and coils used to test the pipe are fixed in RFECT, it is a shortcoming that the practical validation of the discussed method is not provided. The parameters set in the simulation model of PRFECT are obtained by optimizing the parameters of practical RFECT tool. The future work will focus on the modification of the RFECT tool to implement the validation of proposed method, and it would be a huge work. This paper has already provided a good theoretical guidance of preprocessing the signals in practical PRFECT of ferromagnetic pipes.

## Figures and Tables

**Table 1:** Parameters of the coils and pipe set in ANSYS.

Name	Length (mm)	Inner Diameter (mm)	Outer Diameter (mm)	Turns	Resistivity (ohm/m)	Wire Diameter (mm)
Transmitter	167	28.4	44.4	3775	4.247 × 10^−8^	0.58
Sensing coil 1	19.1	26.3	32.7	9275	3.083 × 10^−7^	0.051
Sensing coil 2	19.1	26.3	32.7	9275	3.083 × 10^−7^	0.051
Pipe	2050	153.7	177.1		3.083 × 10^−7^	
